# Trichiniasis

**Published:** 1867-06

**Authors:** 


### ARTICLE III.

### *Trichiniasis.*

Pork eaters have been recently startled by alarming announcements of the danger they run in devouring their favorite viands, and begin to suspect that there may have been some medical reason for the rigorous prohibition of this article of food by the great legislator of the Jews. Under some circumstances this meat has proved as deadly as arsenic or strychnia. The public attention which has been bestowed upon it, is our only apology for deviating so far from our ordinary course as to give a brief account of this curious disease produced by measly pork.

The first note of alarm was sounded from Germany, which country has enjoyed a most unfortunate preeminence in this distressing malady. The severest attack, and that which has most attracted the attention of the unprofessional was that of last October at Hedersleben, where, out of three hundred and fifty who were attacked, eighty died. This severe mortality naturally excited much anxiety all over the world where pork is eaten.

The symptoms of the disease are peculiar, and until recently so obscure as to lead to the confounding it with other maladies, such as inflammatory rheumatism, typhoid fever and even Asiatic cholera. Generally the earliest indications are those of gastro-intestinal irritation, arising from the presence of the parasite in the alimentary canal. Vomiting, diarrhœa, violent colic, loss of appetite, and general distress, reaching sometimes to the agony of incipient cholera, ushers in the attack. Headache follows, and then comes on a general swelling of the face. Red watery eyes look timidly from between their swollen lids, dreading any intensity of light. The patient rolls his eye-balls with pain, and the swelling increases till the whole face is bloated. The tongue thickens, and the dropsical glottis gives out harsh and husky tones. The neck hardens, and the corded veins stand out upon its tightened surface. The tortured sufferer tries in vain to stretch his limbs, for every muscle is swollen and resists pressure like a piece of India rubber. The slightest touch is exquisitely painful and motion becomes impossible. The patient lies on his back, his joints locked in the rigidity of tetanus, and the sweat pouring out in astonishing quantities. Sometimes this sweating continues in one part of the body, occasionally in a single limb, after it has ceased elsewhere. All this while the fever rages violently. After a time, the diaphagm and the intercostal muscles are attacked, they stiffen, the patient pants, his tongue grows dry, his weak thready pulse flutters, and in a low muttering delirium death finally comes, and kindly puts an end to the wearing agony.

All cases, however, are not fatal. The majority of those hitherto described have recovered. When the favourable change takes place there is a gradual improvement in the symptoms. The fever subsides, the sweats diminish, the tenderness of the muscles decreases. A swelling sometimes amounting to a general dropsy sets in. The patient moves his limbs, but feebly, and for a long time walking is very painful. The hair and nails fall off, and the skin sometimes exfoliates in large flakes.

The cause of all this distress is the *trichina spiralis*, a little creature that lies coiled up in a small cyst among the muscles. So long as it remains encysted it is without sex and inactive. As soon however as it is introduced into the intestinal canal, it is warmed into a dangerous vitality. Sexual organs are developed, and energetic reproduction begins. The female which is twice the length of the male brings forth her young alive, and spawns from two hundred to a thousand. Her mischievious career is then run, and she is discharged from the intestinal tract. In two or three days or a week after the indulgence in trichinosed food, these swarms of parasites are hatched, and are ready to set up housekeeping on their own account. They are very minute and require a strong magnifying power to see them clearly. When enlarged, they have somewhat the appearance of an earth-worm. With their sharp pointed head they attack and perforate the walls of the intestinal canal,* working their way straight to their destination, the fibres of the voluntary muscles. As soon as their progress is stopped, they envelop themselves in a capsule, and remain quiet until introduced into the alimentary canal of some other unfortunate creature. Hence persons, who survive the first invasion into their muscles of these swarms of enemies, usually recover, and it has been observed that they are apt to become excessively corpulent. Some con-

*Some observers think they pass along the blood-vessels in the current of the circulation, but the statement of the text has the weight of authority in its favour.

nexion has been suspected between trichinæ and cancer, but this is at present only a suspicion.

Pagentescher and Fuchs studied carefully the relations of different animals to these pestilent invaders, and found great diversities in their susceptibility to this plague. Of the carnivorous animals, the dog is invulnerable, while the cat is easily infected. We have more interest in those creatures which are used for human food. Of these, the rabbit is very susceptible to this living poison, while sheep, oxen, cows, goats, calves and deer are rarely if at all attacked. Birds enjoy entire exemption, and so do fish, reptiles and crabs. The most easily infected of all creatures is the hog, and it is from it that man obtains these pestilent entozoa. Some cases have been apparently traced to beef and veal, but nearly all the recorded patients have been undoubtedly infected by pork. Where the hog gets these parasites remains as yet a mystery. Various vegetables and animals have been suspected, but the *corpus delicti* has not yet been unmistakeably found on any of them.

Wherever generated, we know that these parasities possess a remarkable tenacity of life. They can endure a cold of thirteen degrees below Fahrenheit's zero point, and still retain their mischievous vitality. Chloroform, arsenic, chromate of potash and other energetic agents will not kill them. Salt was at one time thought to be fatal to them, but patients have been infected with hams that had been salted over a month and smoked afterwards. There is but one power that can destroy them, and that is a heat of 170° and upwards which will coagulate their albuminous tissues. Hertwig, however, says that he boiled meat containing these creatures for twenty-two minutes without killing them, but that a further boiling of three minutes deprived them of life.

We have already stated that Germany was the chief sufferer from this disease. It is remarkable that the French neighbours of these unlucky Germans, who eat the same pork, are never attacked. The difference is manifestly due to the different habits of the people. The Frenchman cooks his food, the German is fond of eating it raw. His sausages smoked for a short time furnish a large portion of his daily food. So severe has been the penalty he has been compelled to pay for this highly prized luxury, that some of the governments have made the selling of pork illegal until it has been microscopically examined. Trichina, however, has not been confined to Germany. We have long known it to exist in considerable quantities in Western pork. Deaths have occurred in several instances in the United States, and have been traced directly to ham. In all cases, however, the sufferers were Germans, and they had eaten their meat raw.

These facts show that in order to escape infection, it is necessary to do one of two things, either to abstain wholly from the use of pork, or to see that it is very thoroughly cooked before it comes on our tables.

